# Adjuvant antimicrobial activity and resensitization efficacy of geraniol in combination with antibiotics on *Acinetobacter baumannii* clinical isolates

**DOI:** 10.1371/journal.pone.0271516

**Published:** 2022-07-21

**Authors:** Choon-Mee Kim, Young Jin Ko, Seul-Bi Lee, Sook Jin Jang

**Affiliations:** 1 Premedical Science, College of Medicine, Chosun University, Gwangju, Republic of Korea; 2 Department of Laboratory Medicine, College of Medicine, Chosun University, Gwangju, Republic of Korea; Laurentian University, CANADA

## Abstract

Adjuvant use of geraniol, a plant essential oil component, is known to increase the efficacy of antibiotics by acting as a potent inhibitor of efflux mechanisms. In this study, we assessed the effect of a geraniol–antibiotic combination in 21 *Acinetobacter baumannii* clinical isolates consisting of high efflux (HE) and low efflux (LE) activity groups. We determined the MIC for geraniol and the four antibiotics and evaluated the adjuvant antimicrobial activity and resensitization efficacy of adjuvant geraniol. Geraniol–antibiotic combinations significantly reduced the MIC of all four antibiotics (*P* < 0.0001), and the fold change in MIC decreased by 4 to >256-fold for tigecycline, >16 to >4,096-fold for ceftazidime, 1 to >4,096-fold for cefepime, and >2 to >4096-fold for ciprofloxacin. Importantly, geraniol showed adjuvant antimicrobial activity and resensitization efficacy when used in combination with antibiotics in 21 *A*. *baumannii* clinical isolates. However, there was no statistically significant difference between the HE and LE groups. Low concentrations (0.125% and 0.0625%) of geraniol showed no cytotoxic or hemolytic activity. Our study shows that geraniol, acting as an antibiotic adjuvant, is a good candidate for in vivo studies of combination therapy for the treatment of MDR/XDR *A*. *baumannii* infections.

## Introduction

*Acinetobacter baumannii*, an opportunistic pathogen associated with nosocomial infections, poses major public health problems because it can develop resistance to numerous drugs due to its ability to upregulate or acquire resistance determinants [1]. The prevalence of multidrug-resistant (MDR) or extensively drug-resistant (XDR) *A*. *baumannii* has increased by at least 80% over the past decades [2]. Combination therapy often improves clinical outcomes, reduces the likelihood of developing multidrug resistance, and increases antibiotic effectiveness [3]. Although it is controversial whether combination antimicrobial therapy for MDR pathogen infections is superior to monotherapy, combination antimicrobial therapy has shown excellent results in vitro, with various antibiotics acting synergistically [4–6].

There have been many studies on the antimicrobial activity of antibiotics combined with essential oils [7]. Geraniol is an acyclic monoterpene alcohol that is found in essential oil from lemongrass, lime, ginger, lavender, etc. Geraniol is used as a flavoring agent in foods and is approved by the United States Food and Drug Administration (FDA) [8]. Geraniol has antimicrobial, antioxidant, anti-inflammatory, and antitumor activities [9]. Particularly, geraniol is known to have a synergistic effect when combined with other drugs and to increase the efficacy of antibiotics by acting as a potent inhibitor of efflux mechanisms. The use of geraniol in combination with antibiotics may expand the treatment options for available antimicrobial agents [10–12]. Antibiotic adjuvants potentiate antibiotic activity when used in combination with antibiotics by changing the physiology of resistant cells [13]. Antibiotic adjuvants have the potential to act as complementary therapy to antibiotic treatment, extending the lifespan of antibiotics and reducing additional resistance, thereby resensitizing bacteria to antibiotics (making resistant bacteria susceptible again) [14,15]. Antibiotic adjuvants may be promising candidates for antibiotic combination therapy, and there is a need to develop effective antibiotic adjuvants that can be used to treat MDR/XDR *A*. *baumannii* infections.

In our previous study, we analyzed the relationship between efflux activity and antibiotic resistance by dividing 120 *A*. *baumannii* clinical isolates into susceptible group and non-susceptible group for each of 16 antibiotics. As a result, it was found that four antibiotics (tigecycline, ceftazidime, cefepime, and ciprofloxacin) were profoundly affected by efflux activity (*p* value < 0.0001) [16]. Therefore, we selected these four antimicrobial agents as the testing antibiotics and used them for evaluation study for the efficacy of antibiotic adjuvants of geraniol, which is known as a potent inhibitor of efflux mechanisms.

In this study, we determined the minimum inhibitory concentration (MIC) of tigecycline, ceftazidime, cefepime, and ciprofloxacin and investigated the effect of geraniol–antibiotic combinations on their MICs in *A*. *baumannii* clinical isolates. Moreover, we assessed the adjuvant antimicrobial activity and resensitization efficacy according to geraniol addition and investigated whether geraniol acts as an efflux pump inhibitor using 21 *A*. *baumannii* clinical isolates with either high efflux (HE) or low efflux (LE) activity.

## Materials and methods

### Bacterial strains and drugs

A total of 21 *A*. *baumannii* strains were used in this study. The *A*. *baumannii* strain ATCC19606 was used as a reference. Twenty *A*. *baumannii* clinical isolates were selected based on the relative expression levels of the resistance-nodulation-cell division (RND) efflux pump *adeB* gene and Hoechst 33342 (H33342) efflux activity from 120 strains in our previous study [17]. These clinical isolates were collected from January 2012 to December 2015 at Chosun University Hospital and were isolated from sputum (12 isolates), open pus (4 isolates), whole blood (2 isolates), catheterized urine (1 isolate), and central venous catheter tip (1 isolate), respectively. Antimicrobial susceptibility testing for the antimicrobial agents routinely tested for *Acinetobacter* species was performed using an AST-N225 card with the VITEK 2 system (bioMérieux, Marcy l’Etoile, France) according to the manufacturer’s recommendations (S1 Table) [18]. Geraniol, tigecycline, ceftazidime, cefepime, and ciprofloxacin were purchased from Sigma-Aldrich (St. Louis, MO, USA).

### Measurement of efflux activity using the relative expression of the RND efflux pump *adeB* gene and H33342 accumulation ratio

We performed real-time reverse transcription PCR to measure the relative expression level of the RND efflux pump *adeB* gene [17]. The *rpoB* gene was used as an internal control for normalization of the *adeB* gene using the ΔΔCt method. The normalized expression of the *adeB* gene was further calibrated with that of *A*. *baumannii* ATCC 19606, which was assigned a value of 1.0. All experiments were performed in triplicate.

Additionally, we performed the H33342 accumulation assay to assess efflux activity [17]. Logarithmic phase cells were incubated with 2.5 μM H33342 with or without the efflux pump inhibitor CCCP, and then fluorescence was measured at steady state (50 min). The H33342 accumulation ratio was calculated by dividing the amount of accumulated H33342 in the presence of CCCP by the amount of accumulated H33342 in the absence of CCCP. The H33342 accumulation ratio was normalized to that of *A*. *baumannii* ATCC 19606, which was assigned a value of 1.0. The normalized H33342 accumulation ratio was expressed as H33342 efflux activity.

### Determination of the MICs for geraniol and the four antibiotics

The MICs for geraniol and ciprofloxacin were determined by the broth dilution method, and those for tigecycline, ceftazidime, and cefepime were determined by the agar dilution method as described by the Clinical and Laboratory Standards Institute [18]. Antibiotic concentrations ranging from 256 to 0.0625 μg/mL were used for MIC determination and the geraniol–antibiotic combination study. Geraniol at concentrations ranging from 32% to 0.125% was used for MIC determination. For MIC determination using the broth dilution method, each agent was added to Muller–Hinton (MH) broth (BD, USA) with bacterial inoculums of 5 × 10^5^ CFU/mL, and incubated at 37°C for 18–20 h. For MIC determination using the agar dilution method, each *A*. *baumannii* strain with a final inoculum of 10^4^ CFU/spot was inoculated on MH agar (BD, USA) containing antibiotics and incubated at 37°C for 18–20 h. The MIC was recorded as the lowest concentration of agents that showed no visible growth.

A geraniol–antibiotic combination study was performed by the broth dilution or agar dilution method as described above. We used geraniol at 0.125%, which is 1/4 of the lowest MIC obtained for the 21 *A*. *baumannii* strains. Each *A*. *baumannii* strain was inoculated on MH broth or MH agar containing antibiotics alone or in combination with geraniol.

We investigated the adjuvant antimicrobial activity and resensitization efficacy of geraniol in combination with an antibiotic. We considered that geraniol had an adjuvant antimicrobial activity when the MICs of antibiotics decreased by more than 4-fold upon geraniol addition. Additionally, we considered that geraniol had resensitization efficacy when resistant clinical isolates were converted to susceptible upon geraniol addition in the antibiotic susceptibility test.

We used the following MIC susceptibility breakpoints to determine resistance/susceptibility to each antibiotic in *A*. *baumannii*: tigecycline susceptible (≤2 mg/L), intermediate (4 mg/L), and resistant (≥8 mg/L); ceftazidime susceptible (≤8 mg/L), intermediate (16 mg/L), and resistant (≥32 mg/L); cefepime susceptible (≤8 mg/L), intermediate (16 mg/L), and resistant (≥32 mg/L); and ciprofloxacin susceptible (≤1 mg/L), intermediate (2 mg/L), and resistant (≥4 mg/L) [18,19].

### In vitro cytotoxicity assay

We used Vero E6 cells (African green monkey kidney epithelial cells) and L929 cells (Mouse fibroblast cells). These cells were cultivated in DMEM supplemented with 10% FBS and 1× penicillin/streptomycin at 37°C and 5% CO_2_. Cytotoxicity tests were performed in triplicate and were determined based on lactate dehydrogenase (LDH) release using a CytoTox96 Non-radioactive Cytotoxicity assay kit according to the manufacturer’s instructions (Promega, Madison, WI, USA).

Vero E6 and L929 cells were seeded on 24 plates at 1 × 10^5^ cells/well, incubated for 24 h at 37°C, and washed with serum- and antibiotic-free DMEM. The cells were then exposed to geraniol for 1 h (37°C) at the following concentrations: 0.0625%, 0.125%, 0.25%, 0.5%, 1%, and 2%. Sample supernatants (50 μL) were transferred to a fresh 96-well plate and reacted with CytoTox 96^®^ Reagent (50 μL) for 30 min in the dark. The enzymatic assay was stopped by adding 50 μL of stop solution, and the absorbance was measured at 490 nm using an Epoch^TM^ microplate spectrophotometer (BioTek, Winooski, USA). Cytotoxicity (%) was calculated according to the manufacturer’s guidelines.

### In vitro hemolytic assay

Geraniol (1 mL) at concentrations ranging from 64% to 0.125% was mixed with 1 mL of a 1% human red blood cell (RBC) suspension prepared in PBS containing 1 mg/mL bovine serum albumin (BSA), and then incubated at 37°C for 1 h. The mixture was centrifuged at 2,500 rpm for 5 min at 4°C, and the absorbance of the supernatant was measured at 540 nm using an Epoch^TM^ microplate spectrophotometer (BioTek).

The in vitro hemolytic assay was performed in triplicates. The 0.5% RBC suspension was used as a negative control, and 0.5% RBC suspension treated with 0.02% Triton X-100 was used as a positive control. Hemolysis (%) was calculated by multiplying the OD_540_ of the supernatants by 100. Whole blood donors provided written informed consent for the use of blood samples at the time of donation.

### Statistical analysis

A proportion analysis was performed to determine whether there was a significant difference in adjuvant antimicrobial activity and resensitization efficacy between HE (n = 10) and LE (n = 11) groups or among antibiotics. Differences in MICs between the HE and LE groups before geraniol addition were analyzed using an independent t-test. In addition, the two groups before (n = 21) and after (n = 21) geraniol addition to each antibiotic were analyzed by paired t-test. Proportion analysis was performed using MedCalc software (version 18.11.6; MedCalc Software, Ostend, Belgium). Statistical significance was set at *P* < 0.05.

## Results

### Determination of MIC in *A*. *baumannii* clinical isolates with HE or LE

The 21 strains of *A*. *baumannii* clinical isolates were divided into two groups (HE and LE) based on the relative expression levels of *adeB* and H33342 efflux activity. The mean ± SD of the *adeB* expression in the HE and LE groups was 18.14 ± 3.87 (95% confidence interval [CI]: 15.37–20.90) and 2.64 ±1.89 (95% CI: 1.37–3.1), respectively. The mean ± SD of the H33342 efflux activity in the HE and LE groups was 2.01 ± 0.55 (95% CI: 1.62–2.40) and 0.83 ± 0.08 (95% CI: 0.78–0.89), respectively (Table 1).

**Table 1:** Determination of minimum inhibitory concentration in two groups of *Acinetobacter baumannii* clinical isolates with high and low efflux activity classified based on relative expression of the efflux pump *adeB* gene and H33342 efflux activity*.

Group	Clinical isolates	Anatomical sites	Antibiotic susceptibility	*adeB* expression	HEA	Geraniol MIC (%)	Tigecycline MIC (μg/mL)	Ceftazidime MIC (μg/mL)	Cefepime MIC (μg/mL)	Ciprofloxacin MIC (μg/mL)
HE	160–92	Sputum	XDR	18.35	2.38	2	16	64	256	256
HE	171–84	Sputum	MDR	19.28	2.28	4	16	128	256	256
HE	172–96	Central venous catheter tip	XDR	16.47	1.86	2	8	128	256	256
HE	174–93	Sputum	XDR	14.18	1.56	0.5	8	256	256	256
HE	174–99	Sputum	MDR	16.86	0.90	0.5	16	128	128	256
HE	175–69	Open pus	XDR	14.73	2.47	0.5	8	64	128	256
HE	181–67	Sputum	MDR	27.29	1.81	2	16	128	128	256
HE	189–97	Sputum	XDR	21.00	2.86	2	16	64	128	256
HE	191–52	Sputum	XDR	15.05	2.15	2	16	256	128	256
HE	195–35	Sputum	MDR	18.15	1.87	16	8	>256	0.125	0.125
Mean ± SD of the HE group (95% CI)				18.14 ± 3.87 (15.37–20.90)	2.01 ± 0.55 (1.62–2.40)	3.15 ± 4.64 (-0.17–6.47)	12.80 ± 4.13 (9.85–15.76)	172.80 ± 138.40 (73.78–271.80)	166.40 ± 86.37 (104.60–228.20)	230.40 ± 80.91 (172.50–288.30)
LE	151–36	Sputum	Susceptible	0.87	0.80	0.5	0.25	64	2	0.125
LE	151–71	Open pus	XDR	4.20	0.92	0.5	8	32	128	256
LE	154–61	Sputum	XDR	4.49	0.79	0.5	8	32	128	256
LE	159–86	Whole blood	XDR	1.41	0.71	4	4	256	256	256
LE	162–83	Whole blood	XDR	0.13	0.81	2	8	128	256	256
LE	177–89	Sputum	XDR	1.74	0.91	2	8	256	128	256
LE	182–75	Open pus	Susceptible	5.72	0.78	1	8	64	8	0.5
LE	208–29	Open pus	Susceptible	4.94	0.81	0.5	8	1	2	0.125
LE	209–72	Catheterized urine	Susceptible	2.58	0.76	0.5	8	8	8	0.125
LE	229–15	Sputum	Susceptible	1.94	0.86	2	8	8	4	0.125
LE	ATCC 19606	Reference strain		1	1	1	2	16	256	1
Mean ± SD of the LE group (95% CI)				2.64 ± 1.89 (1.37–3.1)	0.83 ± 0.08 (0.78–0.89)	1.32 ± 1.10 (0.58–2.06)	6.39 ± 2.89 (4.45–8.33)	78.64 ± 94.90 (14.88–142.40)	106.90 ± 109.60 (33.26–180.60)	116.50 ± 133.50 (26.85–206.20)
Mean ± SD of total isolates (95% CI)				10.02 ± 8.45 (6.17–13.86)	1.40 ± 0.71 (1.07–1.72)	2.19 ± 3.34 (0.67–3.71)	9.44 ± 4.76 (7.28–11.61)	123.50 ± 124.30 (66.90–180.10)	135.20 ± 101.50 (89.06–181.40)	170.80 ± 123.50 (114.50–227.00)

*HE, group of *A*. *baumannii* clinical isolates with high efflux activity; LE, group of *A*. *baumannii* clinical isolates with low efflux activity; HEA, H33342 efflux activity; MIC, minimum inhibitory concentration; 95% CI, 95% confidence interval; SD, standard deviation.

The MIC range of geraniol for the 21 clinical isolates was 0.5–16%, and 0.125% geraniol was used for combination studies. The MIC ranges of tigecycline, ceftazidime, cefepime, and ciprofloxacin were 0.25–16, 1–256, 0.125–256, and 0.125–256 μg/mL, respectively. The MICs of tigecycline, ceftazidime, and ciprofloxacin between the HE and LE groups before geraniol addition were statistically different (*P* = 0.0203, *P* = 0.0191, *P* = 0.0369). However, there was no statistical difference in the MICs of cefepime between the two groups (*P* = 0.3252) (Table 1).

### Adjuvant antimicrobial activity and resensitization efficacy of geraniol added to antibiotics in *A*. *baumannii* clinical isolates

The addition of 0.125% geraniol significantly reduced the MIC of antibiotics in 83 out of 84 antibiotic/isolate combinations (98.8%) derived from 21 clinical isolates and four antibiotics (Tables 2 and 3).

**Table 2:** Adjuvant antimicrobial activity of geraniol in two groups of *Acinetobacter baumannii* clinical isolates with high efflux activity and low efflux activity against tigecycline, ceftazidime, cefepime, and ciprofloxacin*.

		Tigecycline MIC (μg/mL)		Fold change	RES		Ceftazidime MIC (μg/mL)		Fold change	RES		Cefepime MIC (μg/mL)		Fold change	RES		Ciprofloxacin MIC (μg/mL)		Fold change	RES	
		GR (-)	GR (+)		GR (-)	GR (+)	GR (-)	GR (+)		GR (-)	GR (+)	GR (-)	GR (+)		GR (-)	GR (+)	GR (-)	GR (+)		GR (-)	GR (+)
HE	160–92	16	<0.0625	>256	R	**S**	64	<0.0625	>1024	R	**S**	256	32	8	R	R	256	4	64	R	R
HE	171–84	16	0.125	128	R	**S**	128	<0.0625	>2048	R	**S**	256	8	32	R	**S**	256	0.125	2048	R	**S**
HE	172–96	8	<0.0625	>128	R	**S**	128	<0.0625	>2048	R	**S**	256	2	128	R	**S**	256	<0.0625	>4096	R	**S**
HE	174–93	8	0.25	32	R	**S**	256	0.25	1024	R	**S**	256	2	128	R	**S**	256	0.25	1024	R	**S**
HE	174–99	16	<0.0625	>256	R	**S**	128	<0.0625	>2048	R	**S**	128	1	128	R	**S**	256	0.125	2048	R	**S**
HE	175–69	8	0.25	32	R	**S**	64	<0.0625	>1024	R	**S**	128	2	64	R	**S**	256	0.5	512	R	**S**
HE	181–67	16	<0.0625	>256	R	**S**	128	<0.0625	>2048	R	**S**	128	<0.0625	>2048	R	**S**	256	<0.0625	>4096	R	**S**
HE	189–97	16	4	4	R	I	64	<0.0625	>1024	R	**S**	128	4	32	R	**S**	256	0.5	512	R	**S**
HE	191–52	16	<0.0625	>256	R	**S**	256	1	256	R	**S**	128	2	64	R	**S**	256	1	256	R	**S**
HE	195–35	8	<0.0625	>128	R	**S**	>256	<0.0625	>4096	R	**S**	0.125	<0.0625	>2	S	S	0.125	<0.0625	>2	S	S
LE	151–36	0.25	<0.0625	>4	S	S	64	<0.0625	>1024	R	**S**	2	2	1	S	S	0.125	<0.0625	>2	S	S
LE	151–71	8	<0.0625	>128	R	**S**	32	<0.0625	>512	R	**S**	128	<0.0625	>2048	R	**S**	256	<0.0625	>4096	R	**S**
LE	154–61	8	0.25	32	R	**S**	32	<0.0625	>512	R	**S**	128	<0.0625	>2048	R	**S**	256	8	32	R	R
LE	159–86	4	0.125	32	I	S	256	4	64	R	**S**	256	<0.0625	>4096	R	**S**	256	<0.0625	>4096	R	**S**
LE	162–83	8	<0.0625	>128	R	**S**	128	1	128	R	**S**	256	2	128	R	**S**	256	<0.0625	>4096	R	**S**
LE	177–89	8	<0.0625	>128	R	**S**	256	2	128	R	**S**	128	4	32	R	**S**	256	<0.0625	>4096	R	**S**
LE	182–75	8	<0.0625	>128	R	**S**	64	<0.0625	>1024	R	**S**	8	0.5	16	S	S	0.5	0.125	4	S	S
LE	208–29	8	2	4	R	**S**	1	<0.0625	>16	S	S	2	<0.0625	>32	S	S	0.125	<0.0625	>2	S	S
LE	209–72	8	<0.0625	>128	R	**S**	8	<0.0625	>128	S	S	8	1	8	S	S	0.125	<0.0625	>2	S	S
LE	229–15	8	<0.0625	>128	R	**S**	8	<0.0625	>128	S	S	4	<0.0625	>64	S	S	0.125	<0.0625	>2	S	S
LE	ATCC19606	2	<0.0625	>32	S	S	16	<0.0625	>256	I	S	256	1	256	R	**S**	1	<0.0625	>16	S	S

*HE, group of *A*. *baumannii* clinical isolates with high efflux activity; LE, group of *A*. *baumannii* clinical isolates with low efflux activity; GR, 0.125% of geraniol; fold change, fold change in MIC decrease upon geraniol addition; RES, resensitization; R, resistance; I, intermediate; S, susceptibility; Adjuvant antimicrobial activity of geraniol against tigecycline, ceftazidime, and cefepime was determined by the agar dilution method and adjuvant antimicrobial activity of geraniol against ciprofloxacin was determined by the broth dilution method; MIC breakpoints for antibiotics; tigecycline-susceptible (MIC ≤2 mg/L), -intermediate (4 mg/L), and -resistant (MIC ≥8 mg/L); ceftazidime-susceptible (MIC ≤8 mg/L), -intermediate (16 mg/L), and -resistant (MIC ≥32 mg/L); cefepime-susceptible (MIC ≤8 mg/L), -intermediate (16 mg/L), and -resistant (MIC ≥32 mg/L); ciprofloxacin-susceptible (MIC ≤1 mg/L), -intermediate (2 mg/L), and -resistant (MIC ≥4 mg/L); bold indicates conversion from resistance or intermediate-resistance to susceptibility in antimicrobial sensitivity testing after 0.125% geraniol addition.

**Table 3:** The MIC ranges, mean MIC, MIC_50_, and MIC_90_ values of four antibiotics with or without geraniol*.

	MIC (μg/mL)	TGC	TGC +GR	Fold change	CAZ	CAZ +GR	Fold change	FEP	FEP +GR	Fold change	CIP	CIP +GR	Fold change
HE	MIC ranges	8–16	<0.0625–4	4 to >256	64 to >256	<0.0625–1	256 to >4096	0.125–256	<0.0625–32	>2 to >4096	0.125–256	<0.0625–4	>2 to >4096
	Mean MIC	12.8	1.16	11.0	135.11	0.63	214.5	166.41	6.63	25.1	230.41	0.93	247.8
	MIC_50_	16	<0.0625	>256.0	128	<0.0625	>2048.0	128	2	64.0	256	0.25	1024.0
	MIC_90_	16	0.25	64.0	256	0.25	1024.0	256	8	32.0	256	1	256.0
LE	MIC ranges	0.25–8	<0.0625–2	4 to >128	1 to >256	<0.0625–4	64 to >1024	2–256	<0.0625–4	1 to >4096	0.125–256	<0.0625–8	>2 to >4096
	Mean MIC	6.39	0.79	8.1	78.64	2.33	33.8	106.91	1.75	61.1	116.55	4.06	28.7
	MIC_50_	8	<0.0625	>128.0	32	<0.0625	>512.0	128	0.5	256.0	1	<0.0625	>16.0
	MIC_90_	8	0.25	32.0	256	2	128.0	256	2	128.0	256	0.125	2048.0
Total	MIC ranges	0.25–16	<0.0625–4	4 to >256	1 to >256	<0.0625–4	>16 to >4096	0.125–256	<0.0625–32	1 to >4096	0.125–256	<0.0625–8	>2 to >4096
	Mean MIC	9.44	1	9.4	104.05	1.65	63.1	135.24	4.54	29.8	170.77	1.63	104.8
	MIC_50_	8	<0.0625	>128.0	64	<0.0625	>1024.0	128	1	128.0	256	<0.0625	>4096.0
	MIC_90_	16	0.25	64.0	256	1	256.0	256	4	64.0	256	1	256.0

*MIC, minimum inhibitory concentration; TGC, tigecycline; GR, 0.125% of geraniol; CAZ, ceftazidime; FEP, cefepime; CIP, ciprofloxacin; fold change, fold change in MIC decrease with geraniol addition; MIC_50,_ MIC required to inhibit the growth of 50% of the isolates; MIC_90,_ MIC required to inhibit the growth of 90% of the isolates.

The decreased MIC range of each antibiotic with geraniol addition was <0.0625–4 μg/mL for tigecycline, <0.0625–4 μg/mL for ceftazidime, <0.0625–32 μg/mL for cefepime, and <0.0625–8 μg/mL for ciprofloxacin. The fold change in MIC decrease due to geraniol addition was 4- to >256-fold for tigecycline, >16- to >4,096-fold for ceftazidime, 1- to >4,096-fold for cefepime, and >2- to >4096-fold for ciprofloxacin (Tables 2 and 3). When we analyzed the changes in MICs between before and after geraniol addition, all four antibiotics resulted in statistically significant decreases in MICs upon geraniol addition (*P* < 0.0001) (Table 2).

Geraniol-induced decrease in MIC_50_/MIC_90_ values in the HE and LE groups was >256/64-fold and >128/32-fold for tigecycline, >2,048/1,024-fold and >512/128-fold for ceftazidime, 64/32-fold and 256/128-fold for cefepime, and 1,024/256-fold and >16/2,048-fold for ciprofloxacin, respectively (Table 3).

The proportion of clinical isolates showing adjuvant antimicrobial activity was 100% (21/21) for tigecycline, ceftazidime, and ciprofloxacin, and 95.2% (20/21) for cefepime. In addition, when we compared the adjuvant antimicrobial activity of geraniol between HE and LE groups, the proportion of clinical isolates showing adjuvant antimicrobial activity was 100% for tigecycline, ceftazidime, and ciprofloxacin in both HE and LE groups, but for cefepime, the proportion of clinical isolates was 100% (10/10) in the HE group and 90.9% (10/11) in the LE group (Table 2).

The proportion of clinical isolates showing resensitization (R→S) was 94.4% (17/18) for tigecycline, 100% (17/17) for ceftazidime, 93.3% (14/15) for cefepime, and 85.7% (12/14) for ciprofloxacin. In addition, the proportion of clinical isolates showing resensitization (R→S) in the HE and LE groups was 90% (9/10) and 100% (8/8) for tigecycline, 88.9% (8/9) and 100% (6/6) for cefepime, and 88.9% (8/9) and 80.0% (4/5) for ciprofloxacin, respectively. However, for ceftazidime, the proportion of clinical isolates showing resensitization was 100% in both HE (10/10) and LE (7/7) groups of *A*. *baumannii*.

When we analyzed the difference in the proportion of clinical isolates showing adjuvant antimicrobial activity or resensitization efficacy according to geraniol addition, there was no significant difference among the four antibiotics or between the HE and LE groups (*P* > 0.05) (Table 2).

### Effect of geraniol on cell viability

**Fig 1:**
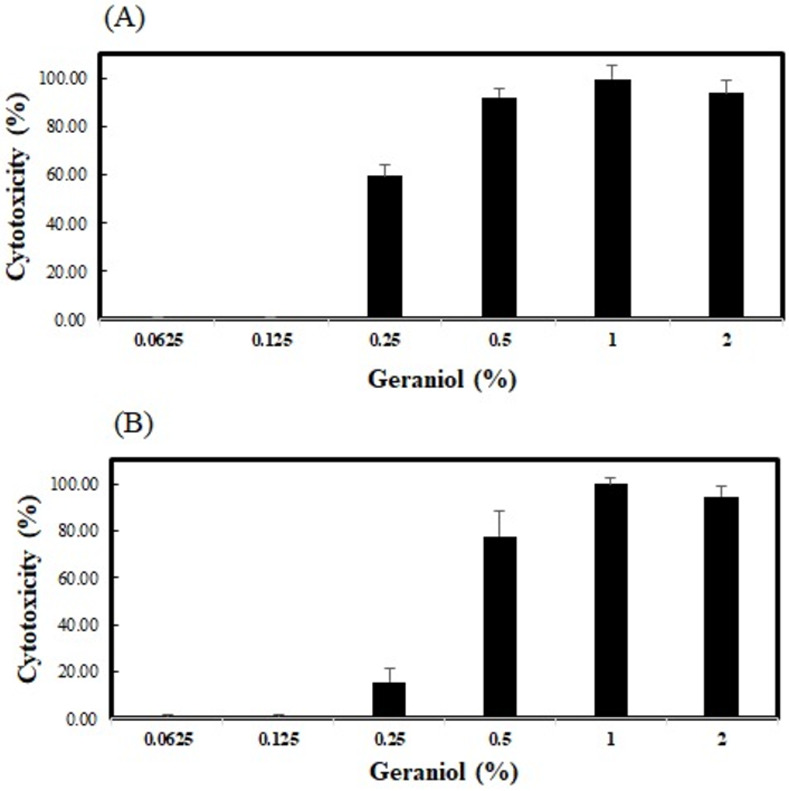
Cytotoxicity activity of geraniol on Vero E6 (A) and L929 cells (B). Vero E6 and L929 cells were exposed to different concentrations (0.0625–2%) of geraniol for 1 h at 37°C, and assessed by the CytoTox 96 Non-Radioactive Cytotoxicity assay. The bars represent means ± SD; n = 3.

We investigated geraniol toxicity on Vero E6 and L929 cells using in vitro cytotoxicity studies. The supernatant obtained from L929 cells (or Vero E6) treated with a high-concentration of geraniol sample solution showed higher absorbance than that obtained from the lysis solution provided in the kit after the enzymatic reaction. Cytotoxicity (%) is expressed as a value relative to the positive control. The group treated with 4% geraniol solution, which showed the maximum LDH release than the lysis solution of the kit, was used as a positive control group, which was considered to have 100% cytotoxicity. The concentration-dependent cytotoxicity of geraniol was observed. The addition of 0.25% geraniol resulted in 15.5% cytotoxicity in L929 cells and 59.5% cytotoxicity in Vero E6 cells. However, 0.125% geraniol showed no cytotoxicity in Vero E6 and L929 cells (Fig 1).

**Fig 2:**
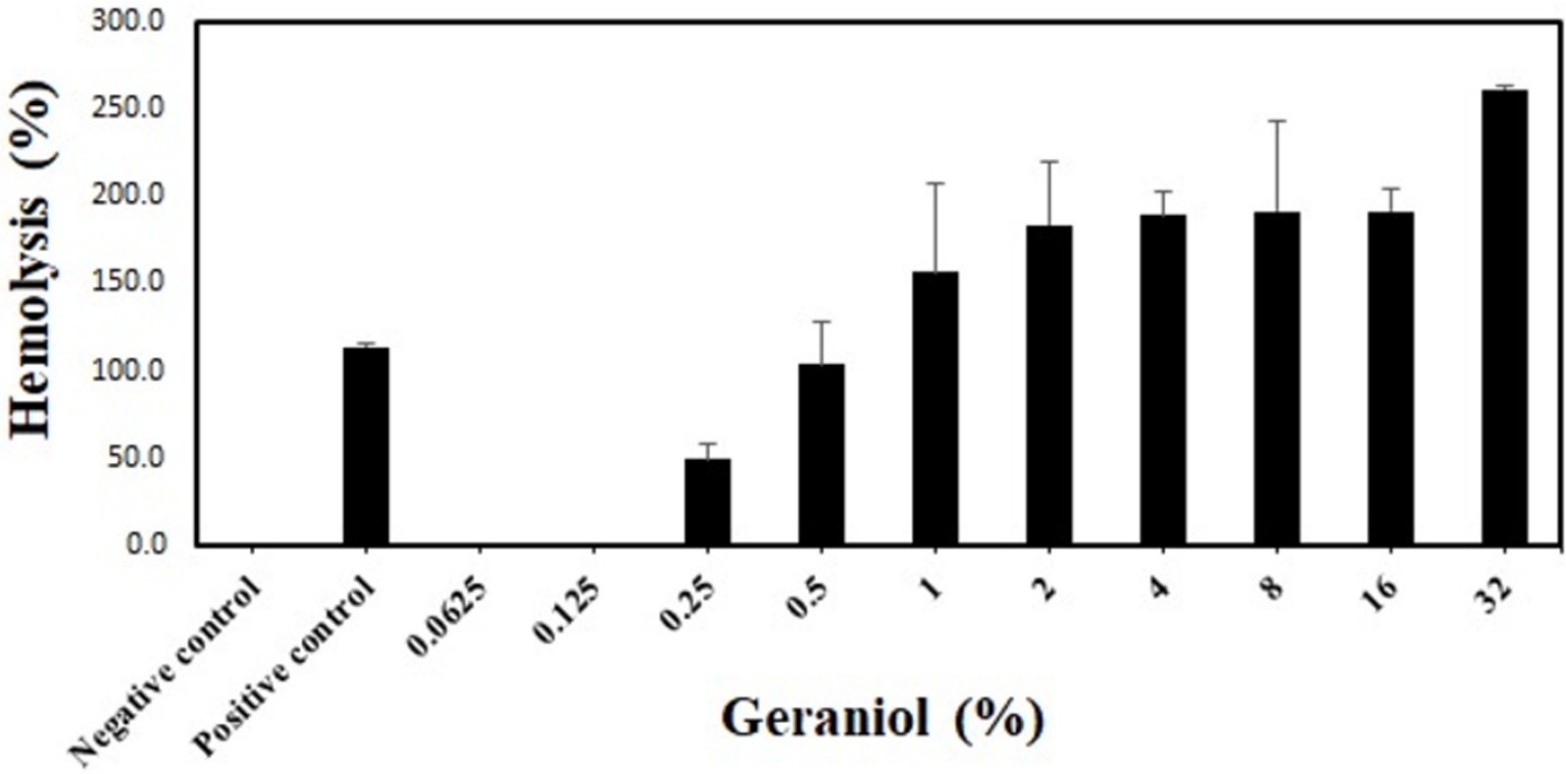
Hemolysis activity of geraniol. The 0.5% RBC suspension was exposed to different geraniol concentrations (0.0625–32%) for 1 h at 37°C, and hemolysis (%) was calculated by multiplying OD_540_ of the supernatants by 100. The bars represent means ± SD; n = 3. PBS, PBS containing 1 mg/mL BSA; negative control, 0.5% RBC suspension; positive control, 0.5% RBS suspension treated with 0.02% Triton X-100.

To confirm the hemolytic activity of geraniol, we performed an in vitro hemolytic assay. Concentration-dependent hemolysis activity of geraniol was observed at more than 0.25% geraniol, which was similar to the cytotoxicity results. However, 0.125% geraniol showed no hemolytic activity (Fig 2).

## Discussion

Essential oils exert antibacterial activity via mechanisms such as destabilization of bacterial membranes, membrane protein damage, proton motive force depletion, and cell contents release. In most cases, essential oils confer antibacterial activity by disrupting the cell wall and membranes, which leads to cell lysis and leakage of cell contents [20]. Geraniol (3,7-dimethyl-octa-trans-2,6-dien-1-ol) is a commercially important terpene alcohol and a major constituent present in the essential oils of several aromatic plants [9]. Geraniol has antimicrobial activity against various bacteria, such as *Pseudomonas*, *Staphylococcus*, and *Escherichia*, and is known to increase the efficacy of antibiotics as it has a synergistic effect when used in combination with other drugs [21,22].

In this study, geraniol–antibiotic combinations significantly reduced the MIC of four antibiotics in 98.8% of total antibiotic/isolate combinations in 21 *A*. *baumannii* clinical isolates compared to a control (antibiotic only); the fold change in MIC decrease due to geraniol was 4- to >256-fold for tigecycline, >16- to >4,096-fold for ceftazidime, 1- to >4,096-fold for cefepime, and >2- to >4096-fold for ciprofloxacin. Lorenzi et al. reported that *Helichrysum italicum* essential oil significantly reduced the chloramphenicol MIC for gram-negative species; specifically, geraniol reduced chloramphenicol resistance of the MDR *Enterobacter aerogenes* strain EAEP289 by as much as 16-fold [10].

The mechanism of the synergistic effect of geraniol–antibiotic combinations is unclear. However, geraniol penetrates into the interior of the bacterial cell and interacts with the intracellular sites, which may have antibacterial potential against various bacteria [21,23]. Vasireddy et al. reported that the antimicrobial activity of geraniol against multidrug-resistant *Burkholderia cepacia* complex isolates is the result of membrane disruption [24].

Factors determining the biological activity of plant essential oils include their composition, functional groups of active components, and synergistic interactions [25]. Guimaraes et al. reported that the compounds with the best activity in both MIC and time-kill kinetics have polar functional groups, which can increase antimicrobial capacity by facilitating penetration through the outer cell membrane [26,27].

Another important mechanism of the synergistic effect of geraniol–antibiotic combinations is the anti-efflux mechanism of geraniol. Geraniol significantly increases the efficacy of β-lactams, quinolones, and chloramphenicol in MDR *E*. *aerogenes* strain EAEP294, which has its *acrAB* operon deleted but still has other active efflux pumps [10,12]. Moreover, Garvey et al. reported that medicinal plant extracts act as inhibitors of efflux for various gram-negative bacteria and show synergistic effects when combined with ciprofloxacin antibiotics [28].

Activation of the efflux pump is an important factor associated with antibiotic resistance in *A*. *baumannii*. Overproduction of the RND efflux pump system with broad substrate specificity is responsible for MDR in *A*. *baumannii* [29]. Among the three members of the *Acinetobacter* drug efflux (Ade) RND system, AdeABC plays an important role in acquired resistance; in particular, the *adeB* gene is widely used for quantification of efflux pump expression as it encodes an essential transporter [29,30].

The fluorescent DNA-binding dye H33342 is a known substrate for RND efflux pumps, thereby making the measurement of H33342 accumulation an efficient approach to assess efflux activity in the MDR phenotype [31,32]. According to our previous study evaluating the correlation between antibiotic resistance and H33342 efflux activity, tigecycline, ceftazidime, cefepime, and ciprofloxacin are antibiotics whose efflux activity significantly affects antibiotic resistance (*P* < 0.000). Therefore, these antibiotics may be good candidates for combination therapy consisting of antibiotics and efflux pump inhibitors [16].

Efflux pump inhibitors, compounds that strengthen antibiotic activity, are effective in rendering MDR gram-negative bacteria susceptible to antibiotics to which they are initially resistant [10,12]. Efflux pump inhibitors, such as PAβN or NMP, have been shown to reduce MICs of antibiotics and reverse multidrug resistance in *A*. *baumannii* isolates [33].

In this study, we investigated whether geraniol acts as a potent efflux pump inhibitor and thus exhibits a synergistic effect with antibiotics. Geraniol–antibiotic combinations significantly reduced the MICs for all four antibiotics, but no difference was observed between clinical strains with HE and LE activity (*P* > 0.05). In addition, adjuvant antimicrobial activity and resensitization efficacy of geraniol did not show a significant difference between the HE and LE groups (*P* > 0.05). These results suggest that MIC decrease, adjuvant antimicrobial activity, and resensitization efficacy of a geraniol–antibiotic combination seem to be the result of mechanisms other than geraniol efflux pump inhibitor activity.

Monoterpenes, such as geraniol, interact with membrane structure proteins due to their lipophilic and solubility properties, resulting in membrane disruption and the antimicrobial effects [23]. Our limitation in this study is that we have not elucidated the potent mechanism of action of geraniol against these *A*. *baumannii* clinical isolates. However, we have shown that geraniol can resensitize bacteria to antibiotics when used in combination with antibiotics. In particular, geraniol clearly possesses antibiotic adjuvant activity and thus enhances antibiotic efficacy; however, further studies are needed to elucidate the synergistic mechanisms. In addition, time-kill study could be necessary to undertake to confirm their bactericidal activity and rate of synergism/indifference/antagonism according to geraniol–antibiotic combination.

Geraniol is considered safe by the FDA. Several studies have shown low mammalian toxicity; in rats, 10,000 ppm geraniol in the diet fed for 16 weeks produced no adverse effects [9,34]. In this study, we confirmed that less than 0.125% geraniol did not show any cytotoxicity in either Vero E6 or L929 cells, as determined by LDH release. However, Queiroz et al. reported that the cell viability of HepG2 cells determined by the MTT test was only 2.23% after a 24-h exposure to 250 μg/mL geraniol; therefore, geraniol showed cytotoxicity even at a low concentration [35]. Cho et al. also reported that geraniol has potent cytotoxic effects against a broad range of cancers, including breast, lung, kidney, and skin cancers, and specifically suppresses tumor growth, not affecting normal physiology at the individual level [34].

In conclusion, geraniol significantly increased antibiotic activity when used in combination with tigecycline, ceftazidime, cefepime, or ciprofloxacin in *A*. *baumannii* clinical isolates. Importantly, geraniol showed adjuvant antimicrobial activity and resensitization efficacy in both *A*. *baumannii* clinical isolates with HE and LE activity. These results indicate that geraniol, as an antibiotic adjuvant, is a good candidate for in vivo studies of combination therapy for MDR/XDR *A*. *baumannii* infections.

## Supporting information

S1 Table Pattern of antibiotic resistance in 20 *Acinetobacter baumannii* clinical isolates used in the study. *GEN, Gentamicin; IPM, Imipenem; MEM, Meropenem; CIP, Ciprofloxacin; TZP, Piperacillin/Tazobactam; SAM, Ampicillin/Sulbactam; TIM, Ticarcillin/Clavulanic acid; FEP, Cefepime; CTX, Cefotaxime; CAZ, Ceftazidime; TMP/SMX, Trimethoprim/Sulfamethoxazole; CST, Colistin; MIN, Minocycline; TGC, Tigecycline.
(DOCX)
