# Supplementary material for: Adjuvant antimicrobial activity and resensitization efficacy of geraniol in combination with antibiotics on Acinetobacter baumannii clinical isolates
Source: PLoS One. 2022 Jul 21;17(7):e0271516. doi: 10.1371/journal.pone.0271516 (PMC9302793; doi:10.1371/journal.pone.0271516)
Supplement: S1 Table — *GEN, Gentamicin; IPM, Imipenem; MEM, Meropenem; CIP, Ciprofloxacin; TZP, Piperacillin/Tazobactam; SAM, Ampicillin/Sulbactam; TIM, Ticarcillin/Clavulanic acid; FEP, Cefepime; CTX, Cefotaxime; CAZ, Ceftazidime; TMP/SMX, Trimethoprim/Sulfamethoxazole; CST, Colistin; MIN, Minocycline; TGC, Tigecycline. (DOCX) [file pone.0271516.s001.docx]

**Supplementary Table 1. Pattern of antibiotic resistance in 20 *Acinetobacter baumannii* clinical isolates used in the study^*^**

| Group | Clinical isolates | Antibiotic susceptibility | GEN | IPM | MEM | CIP | TZP | SAM | TIM | FEP | CTX | CAZ | TMP/  SMX | CST | MIN | TGC |
| --- | --- | --- | --- | --- | --- | --- | --- | --- | --- | --- | --- | --- | --- | --- | --- | --- |
| HE | 160-92 | XDR | R | R | R | R | R | R | R | R | R | R | R | S | S | R |
| HE | 171-84 | MDR | I | R | R | R | R | I | R | R | R | R | S | S | S | R |
| HE | 172-96 | XDR | R | R | R | R | R | R | R | R | R | R | R | S | S | R |
| HE | 174-93 | XDR | R | R | R | R | R | R | R | R | R | R | R | S | S | R |
| HE | 174-99 | MDR | I | R | R | R | R | S | R | R | R | R | S | S | S | R |
| HE | 175-69 | XDR | I | R | R | R | R | R | R | R | R | R | R | S | S | R |
| HE | 181-67 | MDR | I | R | R | R | R | I | R | R | R | R | S | S | S | R |
| HE | 189-97 | XDR | R | R | R | R | R | I | R | R | R | R | R | S | S | R |
| HE | 191-52 | XDR | R | R | R | R | R | R | R | R | R | R | R | S | R | R |
| HE | 195-35 | MDR | R | R | R | S | R | R | R | S | R | R | R | S | S | R |
| LE | 151-36 | Susceptible | S | S | S | S | S | S | S | S | S | R | S | S | S | S |
| LE | 151-71 | XDR | R | R | R | R | R | R | R | R | R | R | R | S | S | R |
| LE | 154-61 | XDR | R | R | R | R | R | R | R | R | R | R | R | S | S | R |
| LE | 159-86 | XDR | R | R | R | R | R | R | R | R | R | R | R | S | S | I |
| LE | 162-83 | XDR | R | R | R | R | R | R | R | R | R | R | R | S | S | R |
| LE | 177-89 | XDR | R | R | R | R | R | R | R | R | R | R | R | S | S | R |
| LE | 182-75 | Susceptible | S | S | S | S | S | S | S | S | S | R | S | S | S | R |
| LE | 208-29 | Susceptible | S | S | S | S | S | S | S | S | S | S | S | S | S | R |
| LE | 209-72 | Susceptible | S | S | S | S | S | S | S | S | S | S | S | S | S | R |
| LE | 229-15 | Susceptible | S | S | S | S | S | S | S | S | S | S | S | S | S | R |

*GEN, Gentamicin; IPM, Imipenem; MEM, Meropenem; CIP, Ciprofloxacin; TZP, Piperacillin/Tazobactam; SAM, Ampicillin/Sulbactam; TIM, Ticarcillin/Clavulanic acid; FEP, Cefepime; CTX, Cefotaxime; CAZ, Ceftazidime; TMP/SMX, Trimethoprim/Sulfamethoxazole; CST, Colistin; MIN, Minocycline; TGC, Tigecycline
